# Impact of Transient and Persistent Acute Kidney Injury on Chronic Kidney Disease Progression and Mortality after Gastric Surgery for Gastric Cancer

**DOI:** 10.1371/journal.pone.0168119

**Published:** 2016-12-09

**Authors:** Chang Seong Kim, Eun Hui Bae, Seong Kwon Ma, Sun-Seog Kweon, Soo Wan Kim

**Affiliations:** 1 Department of Internal Medicine, Chonnam National University Medical School, Gwangju, Korea; 2 Department of Preventive Medicine, Chonnam National University Medical School, Gwangju, Korea; 3 Jeonnam Regional Cancer Center, Chonnam National University Hwasun Hospital, Hwasun-gun, Republic of Korea; Universidade de Sao Paulo, BRAZIL

## Abstract

Acute kidney injury (AKI) is common after gastric surgery for gastric cancer and associated with adverse outcomes. However, the impact of transient or persistent AKI on clinical outcomes after gastric surgery for gastric cancer has not been described. We performed a retrospective study of 4,886 patients with normal renal function who underwent partial or total gastrectomy for gastric cancer between June 2002 and December 2012. AKI patients were classified as transient and persistent AKI based on the return of serum creatinine to the level indicating no AKI within 7 days. Our outcomes included occurrence of new-onset chronic kidney disease (CKD) and mortality 1 year after gastric surgery. AKI occurred in 638 (13.1%) after gastric surgery. Transient AKI was documented in 574 (90%). Use of diuretics and contrast agents was a common risk factor for persistent and transient AKI. Length of intensive care unit (ICU) and hospital stay, and ICU admission rate were higher in patients with transient AKI than in those without AKI. Although patients with persistent AKI had a higher new-onset CKD 1 year after gastric surgery after adjusting for multiple covariates, transient AKI was not associated with new-onset CKD. The 1-year mortality rates were significantly higher in patients with transient and persistent AKI. Not only persistent AKI but transient AKI is associated with increased risk of hospital complications and a significantly higher risk of long-term mortality than patients without AKI after gastric surgery. Moreover, persistent AKI, but not transient AKI, is associated with CKD progression at 1 year.

## Introduction

Acute kidney injury (AKI) is a common complication associated with high morbidity and mortality in hospitalized patients [1]. Postoperative AKI accounts for about 20−40% of all causes of AKI occurring during hospitalization [2]. Postoperative AKI is not only one of the most common postoperative complications but is also associated with in-hospital mortality, long-term mortality up to 10 years after surgery and an increased risk for progression to chronic kidney disease (CKD) and kidney failure [3, 4]. Although AKI after non-cardiac or non-vascular surgery has not been extensively studied, large studies have demonstrated that incidence of AKI after non-cardiac surgery may increase up to 32% [5]. Among the patients who developed AKI after non-cardiac surgery, gastrointestinal surgery procedures represented a large proportion of surgery types. Interestingly, we found that 14.4% of patients with gastric cancer after gastric surgery developed postoperative AKI. Moreover, these patients developing AKI had a higher risk of 3-month mortality after gastric surgery [6].

AKI could be distinguishable in terms of renal recovery from AKI or duration of AKI, and it has been suggested that transient AKI represents a functional reduction by a rapidly reversible decreased glomerular filtration rate (GFR), whereas persistent AKI may reflect established structural renal tubular damage or dysfunction [7]. Recent studies demonstrated that even transient AKI is associated with increased morbidity or mortality in hospitalized patients [7–12]. Therefore, early identification of transient AKI may help provide proper treatment options to patients after surgery. However, distinguishing transient and persistent AKI from AKI as well as the effect of transient or persistent AKI on clinical outcomes after gastric surgery for gastric cancer has not been described.

We hypothesized that transient AKI is common and would influence the short and long-term clinical outcomes in gastric cancer patients undergoing gastric surgery. The present study aimed to assess the incidence and predictive factors of transient and persistent AKI after gastric surgery for gastric cancer, as well as to determine the association between transient or persistent AKI and progression of CKD and mortality.

## Methods

### Ethics Statement

The study protocol was approved by the Institutional Review Board of Chonnam National University Hospital and Chonnam National University Hwasun Hospital, which waived the informed consent, given the observational study design. This study was conducted in accordance with the Declaration of Helsinki guidelines.

### Study design and patient population

We reviewed the electronic medical records and laboratory results of all adult patients who underwent total or subtotal gastrectomy for gastric cancer in Chonnam National University Hospital and Chonnam National University Hwasun Hospital between June 2002 and December 2012. Among the 5,766 patients identified, patients with preoperative estimated GFR <60 mL/min/1.73 m^2^; emergency operation; history of hemodialysis, peritoneal dialysis, or kidney transplantation; CKD; or insufficient data were excluded from our analysis. We also excluded patients who died within 24 h of gastric surgery because their mortality was not associated with postoperative AKI and the data were inappropriate for aim of our study [6].

### Data collection and Definitions

Demographic and laboratory data were collected from the medical records: age; sex; heart rate; body mass index; previous or current history of hypertension, diabetes mellitus, chronic obstructive pulmonary disease (COPD), and smoking; tumor node metastasis (TNM) stage; levels of serum creatinine, hemoglobin, and albumin; and use of diuretics, non-steroidal anti-inflammatory drugs (NSAIDs), packed red blood cell (RBC) transfusion, contrast agent, and vasopressors. Cases of death were ascertained by data linkage to the national death certificate database of Statistics Korea and the regional cancer registries.

AKI was defined according to the Kidney Disease Improving Global Outcomes (KDIGO) clinical practice guidelines [13, 14]. We used the creatinine criteria only because retrospectively collected urine data have the potential to be inaccurate in this regard. Baseline serum creatinine was based on the most recent value before admission or at admission if former was not available. Estimated GFR was calculated using the Chronic Kidney Disease Epidemiology Collaboration (CKD-EPI) equation [15]. Transient AKI was defined as serum creatinine values returning to the no AKI range within the first 7 days after gastric surgery. Persistent AKI was defined as incomplete reduction of serum creatinine, still classified as AKI irrespective of stage 7 days after gastric surgery. This cutoff was chosen because the majority of the patients with transient AKI demonstrated recovery within 7 days and it may allow for comparison with previous studies in this field [7, 8].

New-onset CKD was defined as a decrease in the estimated GFR to <60 mL/min/1.73 m^2^ after gastrectomy in patients with a preoperative estimated GFR >60 mL/min/1.73 m^2^. Tumor stage was assessed according to the TNM system of the American Joint Committee on Cancer/Union for International Cancer Control [16].

Anemia was defined as hemoglobin level of <13.0 g/dL in men and <12.0 g/dL in women by the World Health Organization diagnostic criteria. Hypoalbuminemia was defined as serum albumin levels of <4.0 g/dL [17]. Intraoperative hypotension was defined as systolic blood pressure of <90 mmHg or use of vasopressors (including dopamine, norepinephrine, epinephrine, and vasopressin) mentioned in the intraoperative anesthesia records as in a previous study [6].

### Statistical analysis

The demographic and biochemical data are expressed as mean ± standard deviation or medians with interquartile (25^th^ and 75^th^ percentiles) ranges for parametric variables and nonparametric continuous variables, respectively. Categorical variables are presented as the number and percentage of patients. We compared variables of the 3 group (no AKI, transient AKI and persistent AKI) by using the Pearson chi-square test and one-way analysis of variance or the Kruskal–Wallis test for categorical or continuous variables, as appropriate. Within-group comparisons for variables were performed using Student’s *t*-test and Mann–Whitney test, or Pearson chi-square and Fisher exact test, as appropriate, followed by a Bonferroni’s correction applied to the post-hoc analysis for multiple comparisons; a *P* value of 0.016 was considered statistically significant [18]. To identify factors that affect transient or persistent AKI, a backward multivariate logistic regression was performed. An analysis of covariance and multiple logistic regressions adjusted to age and sex were performed to evaluate the clinical outcomes (including incidence of renal replacement therapy [RRT], admission rate of intensive care unit [ICU], length of ICU and hospital stay, new-onset of CKD, and death) among groups. Multivariable logistic regression analyses were performed to identify the independent predictors of new-onset of CKD and mortality at 1 year after gastric surgery. Moreover, Cox proportional hazards analysis was performed to investigate independent association between transient or persistent AKI and long-term risk of death in patients undergoing gastric surgery. The following variables required adjustment: age, sex, history of hypertension, diabetes mellitus, COPD, smoking, anemia, hypoalbuminemia, intraoperative hypotension, TNM stage, and type of AKI. The probability of survival was estimated using the Kaplan–Meier method, and curves were compared using the log-rank test among groups. All statistical tests were two-tailed and *P*<0.05 was considered significant. The analyses were performed using the Statistical Package for Social Sciences software, version 21.0 (IBM Corp, Armonk, NY).

## Results

### Baseline characteristics

The mean age of the 4,886 participants was 62.5±12 years; 3,281 (67.2%) were men, and the baseline estimated GFR was 84.5±12.7 mL/min/1.73 m^2^. Among this participants, AKI occurred in 638 (13.1%) after gastric surgery. Of these, transient AKI was documented in 574 (90%) and persistent AKI occurred in 64 (10%) according to our definitions. The recovery period for transient AKI was 1.8±1.3 days. Moreover, 16 patients recovered from persistent AKI over a period of 19.7±24.3 days. The demographic, operative, and biochemical characteristics of the patients with no AKI, transient and persistent AKI are shown in Table 1. Patients with transient AKI were more frequently men, had a higher prevalence of COPD, anemia, hypoalbuminemia, and estimated GFR, longer operation time, greater use of diuretics, contrast agents, and transfusion of packed RBCs than patients without AKI. Compared to the patients with transient AKI, those with persistent AKI had significantly a higher rate of intraoperative hypotension episodes (23.1% versus 39.3%, *P* = 0.007).

**Table 1:** Baseline characteristics.

	No AKI ^a^ (n = 4248)	Transient AKI (n = 574)	Persistent AKI (n = 64)	*P* value
Age	62.4±12.0	63.3±11.9	65.3±11.5	0.040
Male (%)	2791(65.7)	438(76.3)*	52(81.3)*	<0.001
SBP (mmHg)	129±13	130±14	131±16	0.062
DBP (mmHg)	82±17	83±10	83±10	0.460
Heart rate (beats/min)	72±15	73±15	75±18	0.063
Body mass index (kg/m^2^)	23.3±3.2	23.1±3.1	23.3±4.1	0.590
Diabetes (%)	600(14.1)	75(13.1)	11(17.2)	0.606
Hypertension (%) ^b^	1157(27.2)	180(31.4)	25(39.1)	0.016
COPD (%)	188(4.4)	43(7.5)*	6(9.4)	0.001
Smoking (%)	2118(49.9)	315(54.9)	41(64.1)	0.008
Hemoglobin (mg/dl)	13.5±2.0	13.1±2.2*	12.8±2.3	<0.001
Anemia (%) ^c^	1027(24.2)	196(34.1)*	24(37.5)	<0.001
Albumin (g/dl)	3.3(3.1, 3.6)	3.2(3.0, 3.4)*	3.1(2.9, 3.4)*	<0.001
Hypoalbuminemia (%) ^d^	570(13.4)	158(27.6)*	24(37.5)*	<0.001
Baseline creatinine (mg/dl)	0.89±0.16	0.88±0.17	0.87±0.16	0.103
eGFR (ml/min per 1.73m^2^) ^e^	84.2±12.6	86.6±13.2*	86.2±13.6	<0.001
Operation time (hr)	4.02±1.23	4.22±1.26*	4.29±1.48	<0.001
Intraoperative hypotension (%) ^f^	1007(23.8)	131(23.1)	24(39.3)*†	0.016
TNM stage (%)				<0.001
Stage 1	2517(59.3)	285(53.1)	34(60.7)	
Stage 2	478(11.3)	89(16.6)	7(12.5)	
Stage 3	423(11.7)	96(17.9)	8(14.3)	
Stage 4	212(5.8)	67(12.5)	7(12.5)	
Diuretics (%)	1494(35.2)	368(64.1)*	50(78.1)*	<0.001
NSAIDs (%)	74(1.7)	10(1.7)	3(4.7)	0.209
Contrast agent (%)	467(11.0)	146(25.4)*	21(32.8)*	<0.001
p-RBC transfusion (%)	583(13.7)	187(32.6)*	22(34.4)*	<0.001
Postoperative vasopressor use (%) ^g^	275(6.5)	53(9.2)	12(18.8)*	<0.001

^a^ Defined by Kidney Disease: Improving Global Outcomes guideline.

^b^ Defined by a systolic blood pressure of >140 mmHg, a diastolic blood pressure of > 90 mmHg or self-reported hypertension irrespective of anti-hypertensive medications.

^c^ Hemoglobin <13.0 g/dl in men, hemoglobin <12.0 g/dl in women.

^d^ Albumin <4.0 g/dL.

^e^ Estimated GFR, calculated using the Chronic Kidney Disease Epidemiology Collaboration equation.

^f^ Defined by a systolic blood pressure of <90 mmHg or use of vasopressors in the intraoperative anesthesia records.

^g^ Norepinephrine, epinephrine, dopamine, vasopressin, or phenylephrine infusions on postoperative day 1 or 2.

Abbreviations: AKI, acute kidney injury; COPD, chronic obstructive pulmonary disease; TNM, tumor node metastasis; NSAID, non-steroidal anti-inflammatory drugs; p-RBC, packed red blood cell.

* *P* value <0.016 (Bonferonni correction) for comparison between transients AKI and no AKI, or persistent AKI and no AKI.

† *P* value <0.016 (Bonferonni correction) for comparison between persistent AKI and transients AKI.

### Independent predictors of transient and persistent AKI

We identified independent predictors of transient and persistent AKI using a backward multivariate logistic regression analysis (Table 2). Predictors of transients AKI included male sex, history of COPD, lower baseline creatinine, TNM stage, and use of diuretics, contrast agents, transfusion of packed RBCs, and vasopressors. Persistent AKI was significantly associated with male sex, hypoalbuminemia, higher intraoperative hypotension episodes, and use of diuretics and contrast agents. Among these predictors, use of diuretics and contrast agent were common risk factors for persistent and transient AKI after gastric surgery.

**Table 2:** Independent predictors of transient and persistent AKI after gastric surgery (Backward regression).

	Transient AKI ^a^^,^^b^	*P* value	Persistent AKI ^a^^,^^b^	*P* value
Age				
Male (%)	2.12(1.63–2.77)	<0.001	2.03(0.98–4.20)	0.056
Diabetes (%)				
Hypertension (%)				
COPD (%)	1.53(1.03–2.26)	0.035		
Smoking (%)				
Anemia (%)				
Hypoalbuminemia (%)			2.05(1.15–3.65)	0.015
Baseline creatinine (mg/dl)	0.21(0.10–0.43)	<0.001		
Operation time (hr)				
Intraoperative hypotension (%)			2.05(1.16–3.62)	0.013
TNM stage (%)				
Stage 1	Reference			
Stage 2	1.52(1.16–1.98)	0.002		
Stage 3	1.53(1.17–2.00)	0.001		
Stage 4	1.96(1.42–2.71)	<0.001		
Diuretics (%)	2.17(1.78–2.65)	<0.001	4.04(2.04–8.03)	<0.001
Contrast agent (%)	1.51(1.19–1.92)	0.001	1.87(1.02–3.45)	0.044
p-RBC transfusion (%)	1.77(1.41–2.45)	<0.001		
Postoperative vasopressor use (%)	1.64(1.10–2.45)	0.015		

^a^ Odds Ratio (95% confidence interval)

^b^Adjusted for factors included in age, sex, hypertension, diabetes mellitus, COPD, smoking, anemia, hypoalbuminemia, operation time, intraoperative hypotension, TMN stage, the use of diuretics, contrast agent, p-RBC transfusion and vasopressors, and AKI types.

Abbreviations: AKI, acute kidney injury; COPD, chronic obstructive pulmonary disease; TNM, tumor node metastasis; p-RBC, packed red blood cell.

### Clinical outcomes in patients with transient and persistent AKI

Clinical events during the 1-year follow-up period after gastric surgery are listed in Table 3. After adjustment of age and sex, even patients with transient AKI showed a significantly higher ICU admission rate, longer length of ICU and hospital stay, and reflected a higher rate of mortality than those without AKI (in-hospital mortality, 0.1% versus 2.3%, *P*<0.001; 3-month mortality, 0.3% versus 2.8%, *P*<0.001; 1-year mortality, 2.9% versus 7.1%, *P*<0.001). However, there were no significant differences in the new-onset CKD at 3 months and 1 year after gastric surgery between no AKI and transient AKI groups. Meanwhile, patients with persistent AKI showed significantly higher new-onset CKD at 3 months and 1 year as well as longer hospital stay and higher mortality at in-hospital, 3 months, and 1 year time points after gastric surgery than those without AKI or with transient AKI. The causes of 3-month deaths among transient and persistent AKI patients were renal failure in 3 (19%) and 4 (40%), cardiovascular disease in 4 (25%) and 1 (10%), and infection in 5 (31%) and 6 (60%), respectively. Moreover, the causes of 1-year death in transient and persistent AKI were renal failure in 5 (12%) and 6 (50%), cardiovascular disease in 4 (9.7%) and 3 (25%), infection in 12 (29%) and 6 (50%), and malignancy in 7 (17.1%) and 2 (16.7%), respectively. In addition, in transient AKI group, 2 (12.5%) and 13 (31.7%) patients died of unknown causes at 3 months and 1 year, respectively.

**Table 3:** Clinical outcomes according to AKI after gastric surgery.

	No AKI (n = 4248)	Transient AKI (n = 574)	Persistent AKI (n = 64)	*P* value ^a^
RRT or CRRT (%)	0(0)	4(0.7)	7(10.9)†	<0.001
ICU care (%)	42(1.0)	43(7.5)*	9(14.1)*	<0.001
ICU length of stay (days)	0.70±1.89	1.13±8.39*	1.23±3.61	<0.001
Hospital length of stay(days)	11.6±6.7	17.8±19.6*	23.7±23.3*†	<0.001
New-onset CKD (3M) (%) ^b^	109(3.1)	20(4.4)	14(29.2)*†	<0.001
New-onset CKD (1Y) (%) ^b^	219(5.8)	38(7.9)	12(26.1)*†	<0.001
In-hospital death (%)	6(0.1)	13(2.3)*	8(12.5)*†	<0.001
3-months death (%)	11(0.3)	16(2.8)*	10(15.6)*†	<0.001
1-year death (%)	125(2.9)	41(7.1)*	12(18.8)*†	<0.001

^a^ *P* value by age and sex-adjusted analysis of covariance (ANCOVA) or logistic regression as appropriate.

^b^ New-onset CKD was defined as a decrease in the estimated GFR to <60mL(min·1.73 m^2^) after gastric surgery in patients with a preoperative estimated GFR ≥60mL(min·1.73 m^2^).

Abbreviations: AKI, acute kidney injury; RRT, renal replacement therapy; CRRT, continuous renal replacement therapy; ICU, intensive care unit; CKD, chronic kidney disease.

* *P* value <0.016 (Bonferonni correction) for comparison between transients AKI and no AKI, or persistent AKI and no AKI.

† *P* value <0.016 (Bonferonni correction) for comparison between persistent AKI and transients AKI.

### Effect of transient and persistent AKI on long-term kidney function

A total of 269 (6.2%) participants in this study showed new-onset CKD at 1 year after gastric surgery. We performed the multivariate logistic regression analysis to determine the effect of transient and persistent AKI on long-term kidney function in patients undergoing gastric surgery (Table 4). Persistent AKI was significantly associated with the new-onset CKD at 1 year after adjustment for confounding factors (odds ratio [OR], 5.84; 95% confidence interval [CI], 2.76–12.4; *P*<0.001). However, transient AKI showed no significant association with new-onset CKD at 1 year after adjustment (OR, 0.95; 95% CI, 0.64–1.42; *P* = 0.803).

**Table 4:** Independent predictors of 1-year new-onset CKD and 1-year mortality after gastric surgery.

	New-onset CKD ^a^			1-year mortality		
	Odds ratio ^b^	95% CI	*P* value	Odds ratio ^b^	95% CI	*P* value
Age	1.017	1.004–1.030	0.011	1.015	1.000–1.030	0.058
Male	1.057	0.713–1.567	0.783	1.094	0.680–1.761	0.711
Diabetes	1.213	0.833–1.766	0.314	0.841	0.495–1.428	0.522
Hypertension	1.352	0.996–1.835	0.053	0.904	0.607–1.347	0.621
COPD	0.353	0.138–0.904	0.030	1.797	0.910–3.549	0.091
Smoking	1.153	0.801–1.661	0.443	1.060	0.683–1.644	0.795
Anemia	1.196	0.864–1.657	0.281	1.056	0.715–1.560	0.783
Hypoalbuminemia	1.116	0.772–1.614	0.443	1.120	0.732–1.713	0.601
Intraoperative hypotension	1.357	0.988–1.867	0.059	1.063	0.704–1.605	0.772
TNM stage						
Stage 1	reference			reference		
Stage 2	2.765	1.866–4.098	<0.001	3.082	1.733–5.356	<0.001
Stage 3	5.356	3.728–7.659	<0.001	4.293	2.541–7.253	<0.001
Stage 4	7.956	5.155–12.281	<0.001	26.498	16.637–42.203	<0.001
No AKI	reference			reference		
Transient AKI	0.950	0.638–1.415	0.803	1.541	1.021–2.327	0.040
Persistent AKI	5.843	2.764–12.353	<0.001	3.259	1.281–8.288	0.013

^a^ New-onset CKD was defined as a decrease in the estimated GFR to <60mL(min·1.73 m^2^) at 1-year after gastric surgery in patients with a preoperative estimated GFR ≥60mL(min·1.73 m^2^).

^b^ Conditional logistic regression adjusted for factors included in age, sex, hypertension, diabetes mellitus, COPD, smoking, anemia, hypoalbuminemia, intraoperative hypotension, TNM stage and type of AKI.

Abbreviations: CKD, chronic kidney disease; COPD, chronic obstructive pulmonary disease; TNM, tumor node metastasis; AKI, acute kidney injury.

#### Effect of transient and persistent AKI on long-term mortality

**Fig 1:**
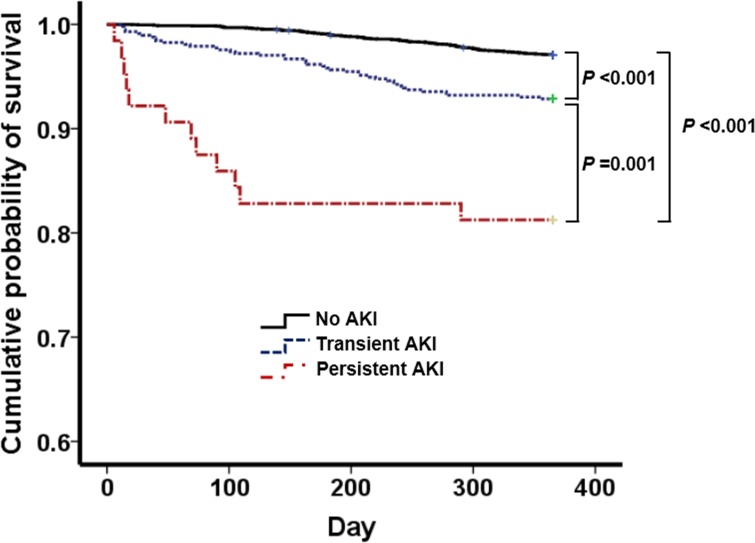
Kaplan–Meier survival curves in patients after gastric surgery for gastric cancer with transient AKI, persistent AKI, and no AKI.

One-year mortality was 178 (3.6%) in the study population. As shown in Table 4, a significant association was found between transient or persistent AKI and 1-year mortality after adjustment. Furthermore, in a multivariable Cox proportional hazards analysis, transient and persistent AKI were an important risk factor for 1-year mortality in patients undergoing gastric surgery even after adjustment for variables (hazard ratio [HR], 1.49; 95% CI, 1.02–2.17; *P* = 0.037; and HR, 2.73; 95% CI, 1.25–5.97; *P* = 0.012, respectively) (Table 5). In addition, Kaplan–Meier curve analysis revealed that patients with transient AKI as well as persistent AKI had significantly higher death rates than those without AKI after gastric surgery (*P*<0.001 and *P* = 0.001, respectively) (Fig 1).

**Table 5:** Prognostic values of transient and persistent AKI for 1-year mortality after gastric surgery (Cox proportional hazards model).

	Unadjusted ^a^	*P* value	Age and Sex adjusted	*P* value	Multivariable adjusted ^a^^,^^b^	*P* value
No AKI	reference				reference	
AKI	2.95(2.14–4.07)	<0.001	2.90(2.10–4.00)	<0.001	1.60(1.12–2.28)	0.009
Transient AKI	2.51(1.76–3.57)	<0.001	2.47(1.73–3.52)	<0.001	1.49(1.02–2.17)	0.037
Persistent AKI	7.46(4.13–13.50)	<0.001	7.23(3.99–13.1)	<0.001	2.73(1.25–5.97)	0.012

^a^ Hazard Ratio (95% confidence interval)

^b^ Adjusted for factors included in age, sex, hypertension, diabetes mellitus, COPD, smoking, anemia, hypoalbuminemia, intraoperative hypotension, TNM stage and type of AKI.

Abbreviations: AKI, acute kidney injury.

## Discussion

The present study showed that most of patients with AKI (90%) undergoing gastric surgery recovered from AKI within the first 7 days after surgery. However, these patients with transient AKI showed significantly higher 1-year mortality than those without AKI. Moreover, we found that patients with persistent AKI had a higher rate of 1-year mortality than those with transient AKI, and were associated with a progression of CKD 1 year after gastric surgery. In addition, use of diuretics and contrast agents were common independent predictive factors for persistent and transient AKI in patients undergoing gastric surgery.

The patients developing AKI are more likely to have poorer short-term mortality after gastric surgery, as shown in previous our study [6]. However, clinical outcomes of early recovery from postoperative AKI is not well evaluated in patients with gastric cancer, despite these patients comprising a relatively large proportion of those developing AKI. Therefore, in the present study, we investigated whether transient AKI after gastric surgery has any clinical implications. Several studies have showed the association between transient AKI and mortality in various disease populations. A recent study showed that transient AKI defined as recovery from AKI at discharge, occurred in 65% of patients with AKI, which was independently associated with 3-year mortality in these patients after acute myocardial infarction [9]. Also, in patients with acute myocardial infarction undergoing percutaneous coronary intervention, contrast-induced AKI patients with transient renal dysfunction, defined as return to baseline creatinine level at 1 month, had a higher 2-year mortality than those without contrast-induced AKI [10]. In addition, a large retrospective study found that patients with transient AKI recovered within 3 days after developing AKI and had a significantly higher hospital mortality compared to patients without AKI [7]. Similar to results of previous studies, our results showed that postoperative transient AKI was a risk factor for 1-year mortality in patients with gastric cancer after adjusting for appropriate covariates. Indeed, compared to those without AKI, patients with transient AKI showing a high rate of ICU admission and longer duration of ICU and hospital stay may be at increased risk for multiple medical complications, which could lead to high risk of mortality even after complete recovery from AKI [12].

Interestingly, recent retrospective studies associating recovery from AKI with long-term mortality after lung transplantation have shown conflicting results. One study demonstrated that patients with completely recovered AKI had a higher long-term mortality than those without AKI [12]. However, another study showed that transient AKI was not associated with long-term mortality in similar population [8]. These discrepancies between two studies might reflect the duration of recovery from AKI according to its own definition of transient AKI. Longer recovery time from AKI during hospitalization might lead to poor long-term mortality than limited time of recovery from AKI, within the 7 days after lung transplantation. Therefore, the relative risk of mortality might also increase as the duration of AKI increased [7]. Indeed, we found that patients with persistent AKI for more than 7 days had a higher risk of long-term mortality than those with transiently early recovery from AKI after gastric surgery.

Several recent studies showed that even AKI with apparent full complete recovery was associated with a loss of renal function in hospitalized patients or in patients undergoing lung transplantation [8, 12, 19, 20]. Particularly, timing of AKI recovery is a strong predictor for development of CKD in patients with recovery from AKI during hospitalization [21]. Although exact pathophysiology are not clearly elucidated, experimental models showed that even with apparent functional recovery form AKI may persist histologic and physiologic changes after AKI and can predispose to long-term CKD risk [22–24]. However, our finding indicated that patients with persistent AKI after gastric surgery were associated with the CKD progression at 1 year, whereas those with transient AKI were not. These different renal outcomes might be involved in various definitions of transient AKI, disease populations and clinical settings among studies [8, 12, 19–21]. Therefore, our findings need to be confirmed in a large multicenter trial with a longer follow-up period.

In the present study, we also identified the independent predictors for transient or persistent AKI. Use of diuretics and contrast agents was independent common predictor for AKI, regardless of recovery status, after gastric surgery. Although it is unclear whether the use of diuretics is a cause or consequence of postoperative AKI, it is clearly associated with increased postoperative AKI, as reported in our previous study [6]. Therefore, indiscriminate use of diuretics to increase urine output in patients with hypovolemic status should be avoided after surgery. Most of the contrast agents were used for enhanced computerized tomography to evaluate complications which included bleeding, infection, or leakage after gastric surgery in our study. Although the pathophysiology of contrast-induced AKI is very complex, it is well known that the use of contrast agents causes renal hypoxia and generation of reactive oxidative stress and has a direct cytotoxic effect on renal tubular epithelial cells [25]. Especially, low level of serum albumin and high intraoperative hypotension episode were associated with persistent AKI in our study. Therefore, identification of patients undergoing gastric surgery who have these predicting factors may allow for the appropriate therapeutic implementation and surveillance, which could significantly improve clinical outcomes.

Our study had several limitations. First, we could not exclude the possibility of residual confounding factors, despite our best efforts to adjust for most confounding factors including TNM stage and operative conditions. Second, the incidence of AKI in our study might have been underestimated, because we used only the change in the serum creatinine to determine the AKI and patients who died within 24 h after gastric surgery were excluded from the analysis. Finally, because the results of our study apply to patients with gastric cancer undergoing gastric surgery, who cannot be generalized into different populations. Despite these limitations, our study included a relative large number of participants and it is the first study to investigate the implication of transient and persistent AKI on clinical outcomes in patients after gastric surgery for gastric cancer.

## Conclusion

Our results showed that patients who recover completely from AKI within the first 7 days after gastric surgery for gastric cancer are at higher risk for long-term mortality than patients without AKI. Moreover, patients who do not recover from AKI are associated with greater long-term mortality and CKD progression then patients who transiently recover from AKI or without AKI. Therefore, we should pay close attention to adverse clinical outcomes and take preventive efforts when AKI occurs after gastric surgery, irrespective of early recovery.
